# Subtle primes of in-group and out-group affiliation change votes in a large scale field experiment

**DOI:** 10.1038/s41598-022-26187-x

**Published:** 2022-12-29

**Authors:** Daniel Rubenson, Christopher T. Dawes

**Affiliations:** 1 Department of Politics, Toronto Metropolitan University, Toronto, M5B 2K3 Canada; 2 Wilf Family Department of Politics, New York University, New York, 10012 USA

**Keywords:** Psychology, Human behaviour

## Abstract

Identifying the influence of social identity over how individuals evaluate and interact with others is difficult in observational settings, prompting scholars to utilize laboratory and field experiments. These often take place in highly artificial settings or, if in the field, ask subjects to make evaluations based on little information. Here we conducted a large-scale (N = 405,179) field experiment in a real-world high-information context to test the influence of social identity. We collaborated with a popular football live score app during its poll to determine the world’s best football player for the 2017–2018 season. We randomly informed users of the nationality or team affiliation of players, as opposed to just providing their names, to prime in-group status. As a result of this subtle prime, we find strong evidence of in-group favoritism based on national identity. Priming the national identity of a player increased in-group voting by 3.6% compared to receiving no information about nationality. The effect of the national identity prime is greatest among individuals reporting having a strong national identity. In contrast, we do not find evidence of in-group favoritism based on team identity. Informing individuals of players’ team affiliations had no significant effect compared to not receiving any information and the effect did not vary by strength of team identity. We also find evidence of out-group derogation. Priming that a player who used to play for a user’s favorite team but now plays for a rival team reduces voting for that player by between 6.1 and 6.4%.

A growing body of empirical evidence suggests we favor individuals belonging to the same social group as us when making a variety of important choices. Shared group identity has been demonstrated to influence who we hire for a job^1^ and choose to work for^2^, are willing to cooperate with^3,4^, and follow^5^. This preference for members of our social group is explained by social identity theory^6,7^—and the closely related self-categorization theory^8^—which holds that an individual’s sense of “who they are” is based in part on their group memberships. These group memberships raise self-esteem by conferring a sense of pride or belonging. Further, we categorize individuals as *us* and *them* based on whether or not they are members of a group, viewing in-group members positively and out-group members negatively^9^. Importantly, since individuals may belong to several social groups, the salience of a particular identity in a given context determines the degree to which it influences behavior^8^.

Since it is difficult to account for all of the attributes that may be correlated with group membership, establishing the causal effect of social identity using observational data has proven to be very challenging. As a result, scholars have utilized laboratory and field experiments to credibly identify in-group and out-group biases. In the laboratory, researchers have studied behavior among subjects assigned to groups based on artificially created^10–13^ as well as existing identities^14–16^. While giving experimenters the ability to establish a causal relationship between group assignment and behavior, a limitation of laboratory experiments is that subjects are placed in a highly artificial environment and thus the results may not extend to more realistic scenarios.

In the field, numerous correspondence and audit studies have tested for discrimination among decision makers in a variety of market contexts by providing information (e.g. resumes) from fictitious individuals^17–20^. Recent studies have extended this work to online platforms such as Craigslist^21^, Amazon Mechanical Turk^2^, eBay^22^ and Airbnb^23^. While correspondence and audit studies are conducted in more realistic conditions, they intentionally limit information about individuals being evaluated. While this is done by design in order to rule out potential confounders, it also creates an artificial information environment that does not speak to settings in which individuals know a great deal about who they are evaluating.

Rather than study in-group favoritism in an artificial or low-information environment, we examine the influence of social identity on decision making in the context of international football where the evaluators, football fans, are highly knowledgeable and those being evaluated, players, are extremely well-known. This context also enables us to test two strong group identities, nationality^24^ and professional team^25–27^. Specifically, we test the degree to which shared and unshared identity influences football fans’ evaluations of professional players. To do so, we partnered with the live score football app *Forza Football* to conduct a large scale field experiment in which participants evaluated the world’s best-known professional football players. Forza Football’s Player of the Season (POS) poll asked users which of 10 candidates was the best player in the world over the previous football season. As part of the POS poll, we randomly informed subjects about the nationality or team membership of POS candidates to test whether priming social identity caused them to support players from the in-group and eschew players from the out-group.

Based on this very subtle prime, we find rather strong evidence of in-group favoritism based on national identity. Priming the in-group status of a player increased in-group voting by 3.6% compared to receiving no information about nationality. Further, the effect of the national identity prime is greatest among individuals who report having a strong national identity. In contrast, we do not find evidence of in-group favoritism based on team identity. Informing subjects of the players’ teams had no significant effect compared to receiving no information and the effect did not vary by strength of team identity. Finally, we also find some evidence of out-group derogation. When we prime supporters of candidates’ previous clubs with the players’ current club affiliation (ie that the player is now a member of the out-group), we find it reduces voting for those players by between 6.1% and 6.4%.

## The experiment

*Forza Football* is a football live score app that at the time of our study had approximately 3.5 million active users around the world. In May of 2018, Forza asked its users to vote in its Player of the Season poll to determine the best player in the world based on the 2017–2018 season. The 2018 ballot consisted of ten players who were from ten different nationalities representing ten different professional teams (see Table 1). Forza Football chose the ten candidates independent of this study. In total, 405,179 votes were cast by Forza users from 35 countries. All methods in this study were carried out in accordance with relevant guidelines and regulations. Forza Football conducted the experiment, which was covered under their user terms and conditions, on the app. Forza provided us with de-identified data (Forza collects no personal information about any of their users) for secondary analysis. Our study was reviewed by the Ryerson University Research Ethics Board (IRB) and deemed exempt (protocol REB 2018-192). Further, since our study involves only secondary use of de-identified data, the Ryerson University Research Ethics Board did not require us get informed consent from Forza users.

**Table 1:** 2017–2018 player of the season candidates.

Player	Nation	Team	Previous team
Neymar da Silva Santos Júnior	Brazil	Paris Saint-Germain	FC Barcelona
Kevin De Bruyne	Belgium	Manchester City	VfL Wolfsburg
David De Gea	Spain	Manchester United	Atletico Madrid
Antoine Griezmann	France	Atletico Madrid	Real Sociedad
Ciro Immobile	Italy	Lazio Roma	Torino FC
Harry Kane	England	Tottenham Hotspur	
Robert Lewandowski	Poland	Bayern Munich	Borussia Dortmund
Lionel Messi	Argentina	FC Barcelona	
Cristiano Ronaldo	Portugal	Real Madrid	Manchester United
Mohamed Salah	Egypt	Liverpool FC	AS Roma

**Figure 1:**
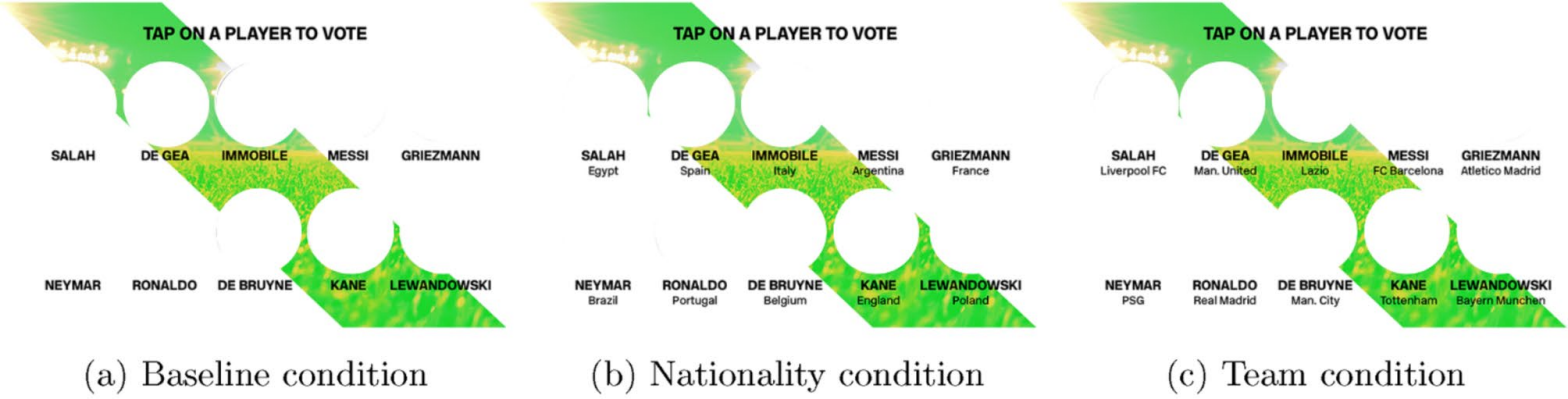
Treatments.

To test the influence of social identity on which player subjects voted for, we randomly manipulated information about the POS candidates shown to subjects in the app. In the condition we refer to as the *nationality condition*, subjects were presented with all ten candidates’ faces, last names, and nationalities. In another condition we refer to as the *team condition*, subjects saw the candidates’ faces, last names, and the professional team they played for. Finally, in the *baseline condition*, subjects were only shown faces and last names. All three conditions are displayed in Fig. 1 (we have removed the photos from Fig. 1 so as to comply with copyright licensing. The player photos used by the Forza Football app were standard headshots of the ten players. For information on how Forza reported on the results of the POS vote, see their blog: https://blog.forzafootball.com/forzafootballplayeroftheseason/).

In the treatment conditions, nationality or professional team information was included in the POS ballot in order to signal to subjects whether the player was a member of their in-group. Therefore, to estimate the effect of primed social identity, we restrict our analysis to subjects who share a group identity (are of the player’s nationality or fans of the player’s team) with one of the POS candidates. All other cases are excluded. More specifically, we estimate the effect of the nationality condition based on subjects from the ten countries listed in Table 1 and the effect of the team condition based on users whose favorite team, as chosen by them in the Forza app, is one of the ten teams listed in Table 1 (Fig. S1 in the Supporting Information provides more details on the various subsamples used for each analysis). For example, a Swedish Forza user would be excluded from the nationality analysis and a supporter of Chelsea Football Club would be excluded from our analysis of the team condition, since there is no Swedish or Chelsea FC players among the ten POS candidates. Nationality was determined based on the country in which the Forza user is located. In the app, users are asked to provide their favorite team in their home country, in each of the the major leagues (Ligue 1, Bundesliga, La Liga, Serie A, and Premier League), and their overall favorite team. We use the team a user chose as their overall favorite.

Imposing this restriction means that in each treatment condition, subjects are informed of a social identity they share with one POS candidate but not the other nine candidates. Thus, one player is a member of their in-group and the rest are members of the out-group. We expect that priming social identity will prompt subjects to vote for players from their in-group since winning the POS poll positively differentiates their team or nation from the others represented in the competition, thus raising their self-esteem^7^.

In order to test for in-group favoritism, we estimate the effect of receiving information priming a shared group identity with a player versus receiving no information. This entails comparing the proportion of subjects voting for in-group players in the nationality/team condition compared to those voting in the baseline condition. Of course, these shared group identities between users and players exist in the baseline condition, but the goal of including the nationality/team information in the treatment condition is to make those group identities salient. Given users in the baseline condition are likely to already be aware of, and possibly thinking about, these player attributes, we did not expect the prime to be particularly strong.

Since social identity theory predicts stronger feelings towards among those with a stronger attachment to the group^28^, we implemented a short in-app survey several weeks *prior* to our study in order measure how strongly subjects identified with their nationality and favorite team. Following^29^, we asked users how strongly they identified with their nationality/football team, whether they say “we” instead of “they” when talking about their nationality / football team, and how important being their nationality/a supporter of their football team was to them (the exact question wording is provided in the Supporting Information, Sect. S2). Possible responses ranged from “not at all” (1) to “extremely” (5). We summed responses from each of the three questions to construct overall measures of national and team identity ranging from 3 to 15. The mean and median value for national identity was 12.3 and 13, respectively and for team identity was 13 and 14, respectively. Since both measures were highly skewed, we created a binary variable denoting strong national and team identity based on whether respondents answered “extremely” for all three identity questions. Based on this classification, 36% of survey respondents reported a strong national identity and 42% had a strong attachment to their favorite football team.

We can also explore the effects of priming out-group identity in the POS poll. In order to do so, we take advantage of the fact that professional football players often change clubs in so-called transfers (the player’s registration is moved from one professional club to another, typically in exchange for a fee). A player who at one point was an in-group member to a supporter of his team becomes a member of that supporter’s out-group when the player moves to a new team. We estimate the effect of receiving the team prime on voting among supporters of the POS players’ *previous* clubs. Doing so makes salient the fact the player was an in-group member but is now an out-group member. For example, Kevin De Bruyne is a Manchester City player in the POS poll but he moved to Manchester City on a transfer from the German Bundesliga team VfL Wolfsburg. Thus, for Wolfsburg supporters, De Bruyne was once an in-group member but when they are primed with information about his playing for Manchester City, he is now an out-group member. As a result, we expect Wolfsburg supporters to be less likely to vote for him in the POS poll compared to the condition in which Wolfsburg supporters do not receive any team information.

For our test of in-group favoritism, we expect the nationality and team conditions to both result in more voting for in-group players than in the baseline condition. We also expect less voting for in-group players compared to the baseline condition when the team condition primes out-group identities. Our hypotheses were pre-registered with the EGAP design registry and our pre-analysis plan can be found at https://bit.ly/33JJfkr.

To test the effects of in-group favoritism, we estimate two-tailed *t*-tests comparing the mean level of voting in the baseline condition to the mean level of voting in the nationality condition and team condition, respectively. We similarly carry out *t*-tests in our evaluation of out-group derogation. To test whether the in-group favoritism elicited by our treatments are conditional on identity strength, we estimate the following linear probability model separately for the nationality condition and team condition:

$$\begin{aligned} Y_i = \beta _0 + \beta _1 Z_i + \beta _2 I_i +\beta _3 Z_i*I_i +\epsilon _i, \end{aligned}$$

where $Y_i$ is whether individual *i* votes for an in-group candidate (0,1), $Z_i$ indicates whether a subject is assigned to the baseline or treatment condition (0,1), and $I_i$ is whether a subject reports having a strong identity (0,1). We are interested in $\beta _3$ which captures the effect of being assigned to the treatment condition and having a strong nationality or team identity. All measures are described in greater detail in the Supplementary Information.

## Results

**Figure 2:**
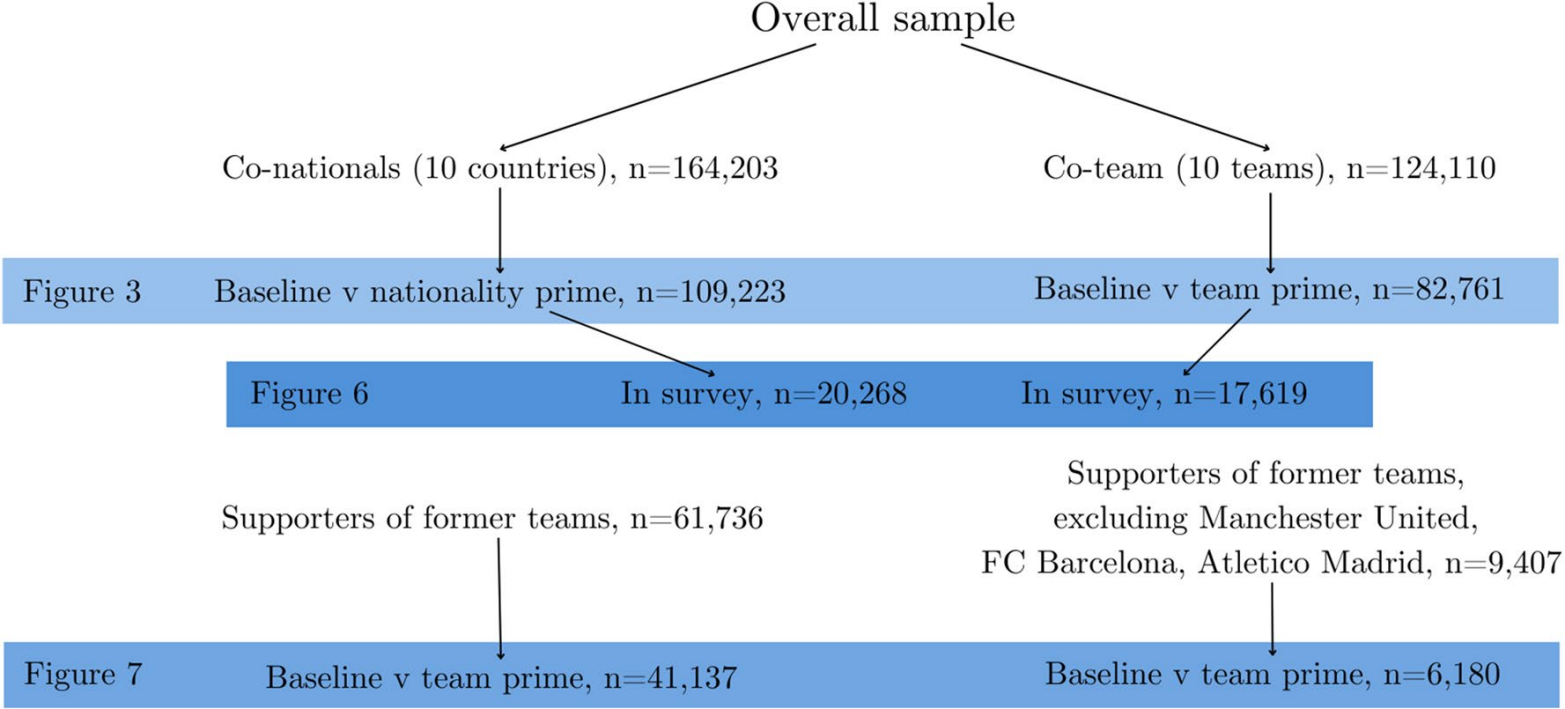
Samples for each analysis.

**Figure 3:**
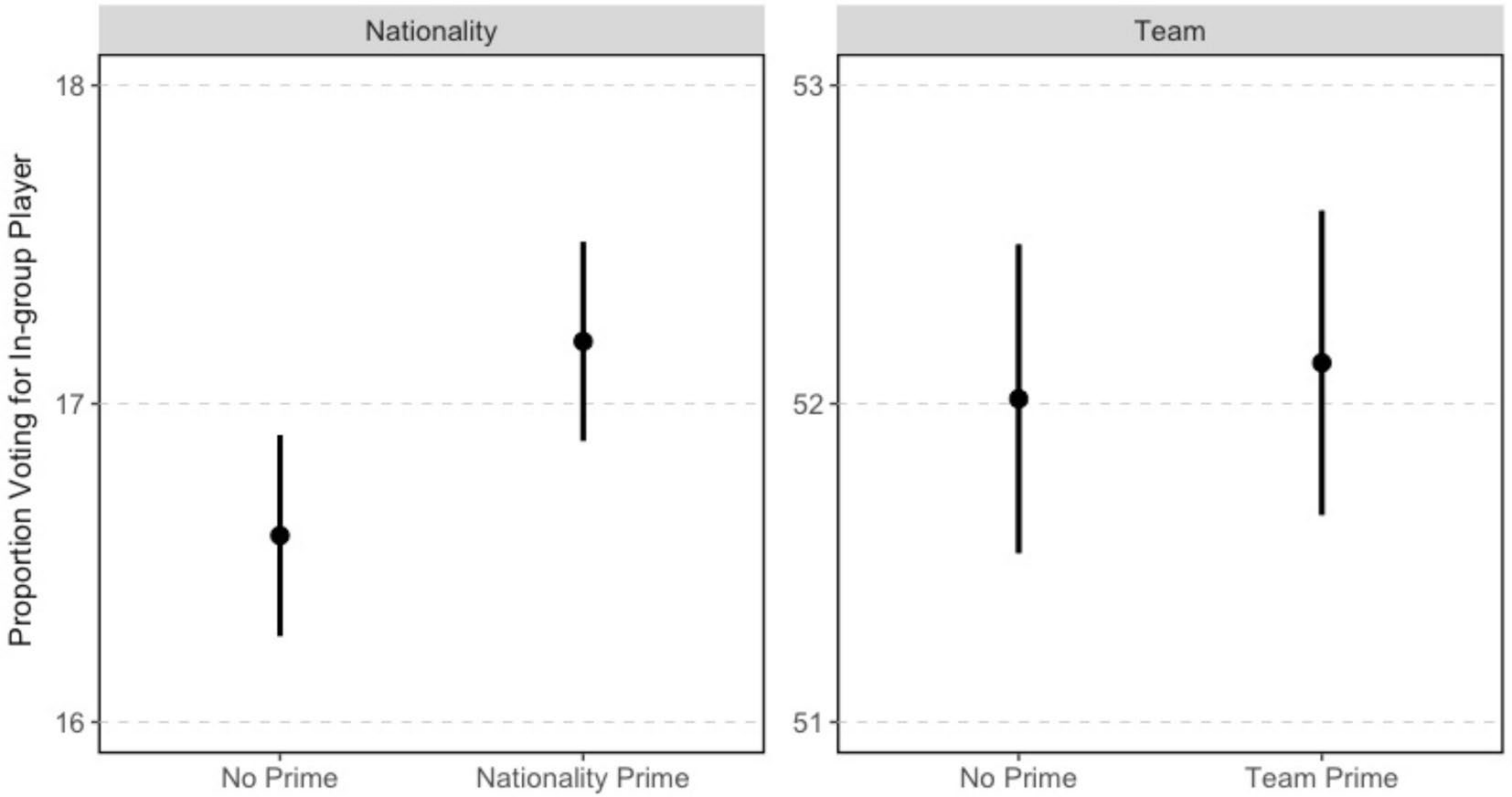
Effect of in-group primes.

Before presenting our results, it is useful to clarify what our sample is for each main analysis we report below. Our sample necessarily differs depending on what comparisons we are analyzing. In Fig. 2 we describe the various cuts to the data that happen at each major analysis we carry out. We begin with the overall sample of Forza users who participated in the POS: 405,179. In Fig. 3, we limit the sample to either co-nationals—users who share nationality with one of the POS candidates—or those who support one of the ten teams of the POS candidates. The former subsample is 164,203, the latter is 124,110. We then compare voting in the baseline condition to that in the nationality condition (or the team condition).

The analysis in Fig. 6 includes those from the above samples who also completed the survey on strength of identity. For Fig. 7 we restrict ourselves first to users who are supporters of former POS candidates’ teams (left panel of Fig. 7) and then further by excluding supporters of Manchester United, FC Barcelona and Atletico Madrid (right panel of Fig. 7).

### In-group favoritism

To evaluate in-group favoritism, we compare voting for in-group players in the baseline condition to the nationality condition ($N = 109{,}223$) and team condition ($N = 82,761$) respectively. Figure 3 displays the mean level of voting and 95% confidence intervals from *t*-tests (all reported *p*-values are two-tailed).

The first thing to note from Fig. 3 is that team is a much stronger predictor of in-group voting than nationality; in the baseline condition (no prime), $16.6\%$ of subjects voted for co-nationals whereas $52.0\%$ voted for a player on their favorite team. This makes sense given that active football fans are typically more interested in, and more often see, players performing for their professional teams than for their national teams.

Turning to our test of in-group favoritism, the nationality prime significantly increased voting for in-group players by 0.6% points ($p < 0.01$) compared to receiving no additional information about the player. The team prime had a small and insignificant effect (0.1% points, $p=0.75$). Taking into consideration the low base rate of voting for co-nationals, the nationality prime caused a 3.7% increase in voting for an in-group player compared to receiving no prime.

**Figure 4:**
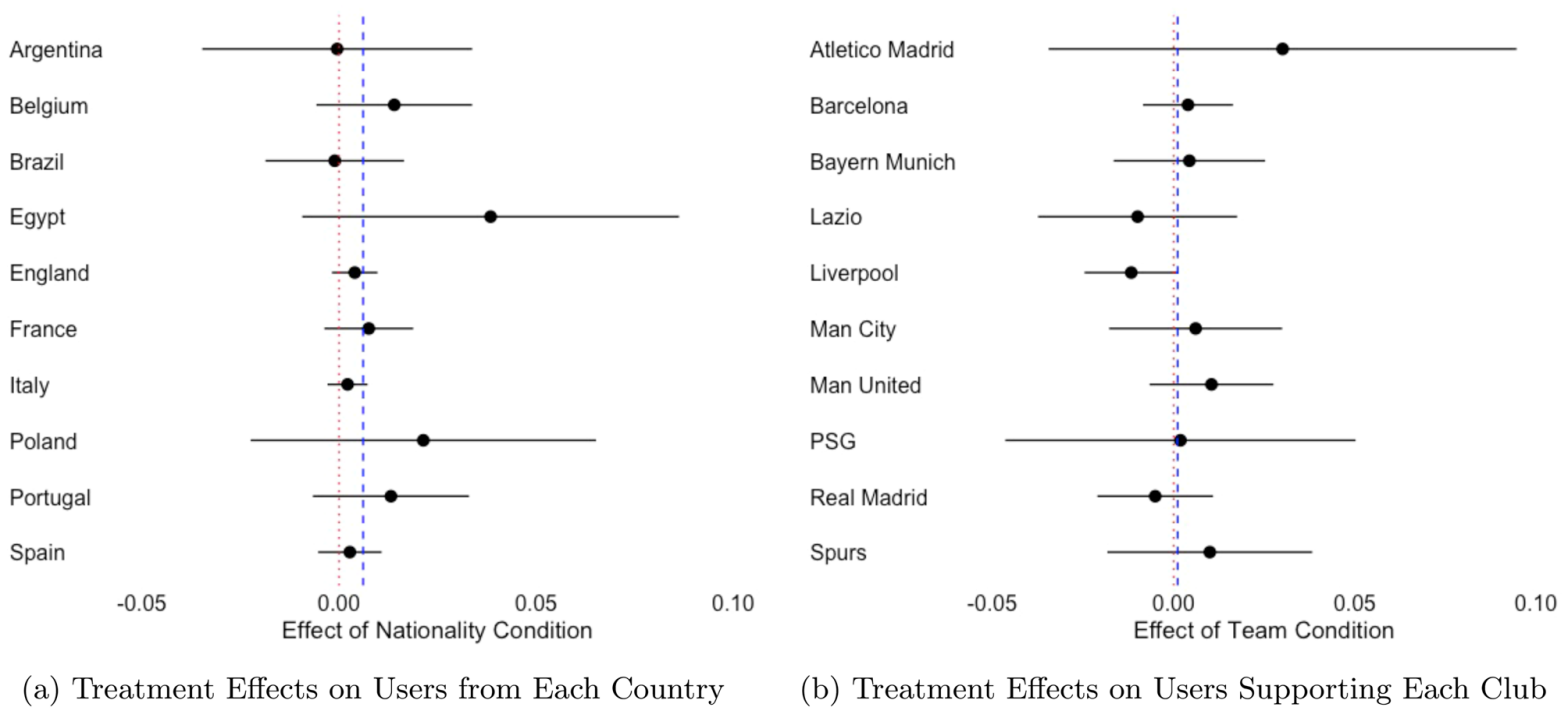
The dots represent the estimated treatment effect with 95% confidence intervals around them. The blue line represents the estimated treatment effect based on the entire sample.

To put the treatment effect in perspective, we can utilize the fact that the final day of the English Premier League season coincided with the second day of the POS poll. On that day, Mohamed Salah broke the Premier League’s single season scoring record by netting his 32nd goal. On the record-setting day, voting for Salah in the POS poll (among those who received the baseline condition) increased by 2.8% (from 47.5% on May 12 ($N=60{,}749$) to 48.8% on May 13 ($N=44{,}585$), $p < 0.01$). A concern might be that these results are due to peculiarities of some countries or clubs. We re-ran the analysis leaving out each country and each club. The results, presented in Fig. 4, indicate our findings are robust to this concern.

**Figure 5:**
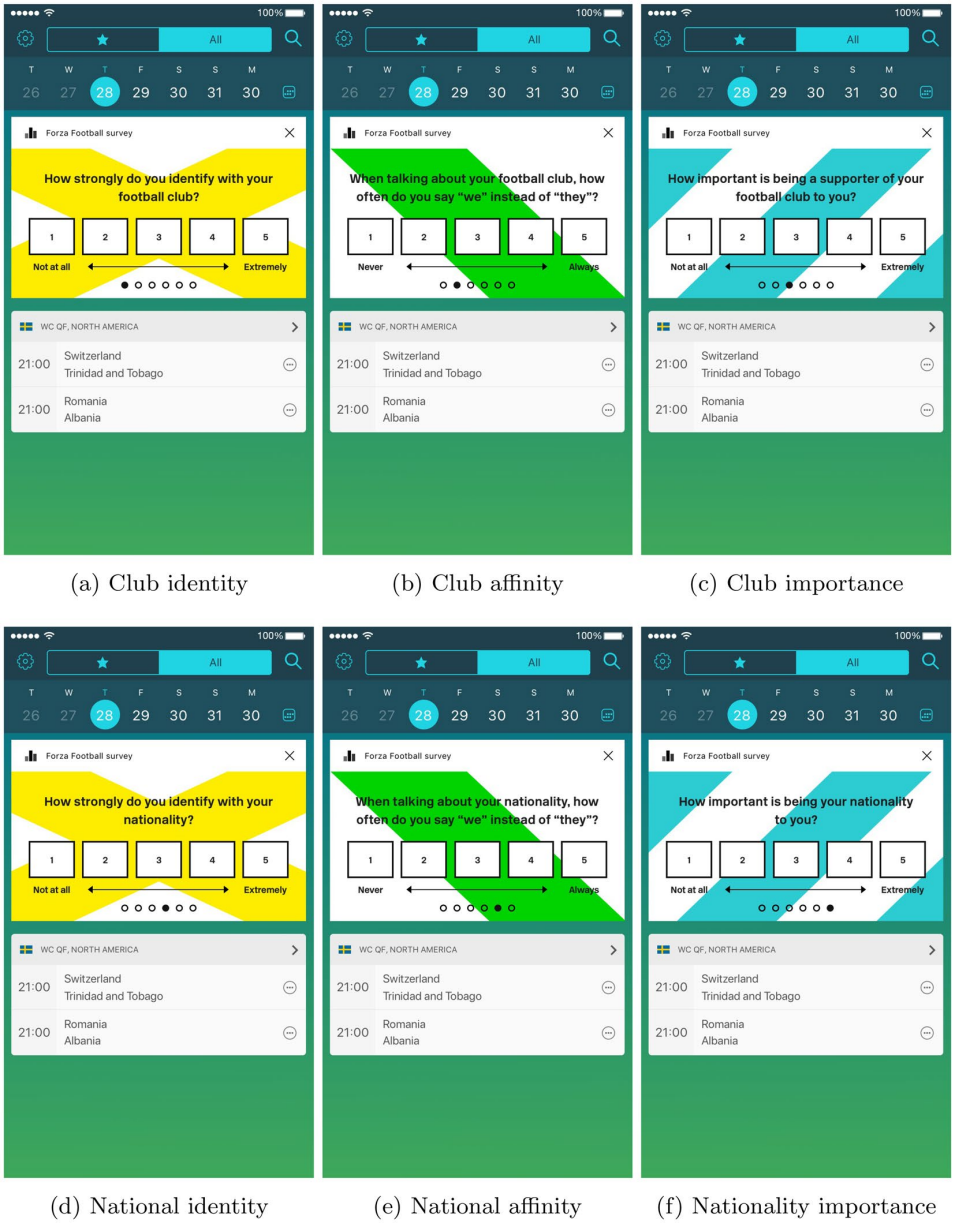
Group identity in-app survey.

**Figure 6:**
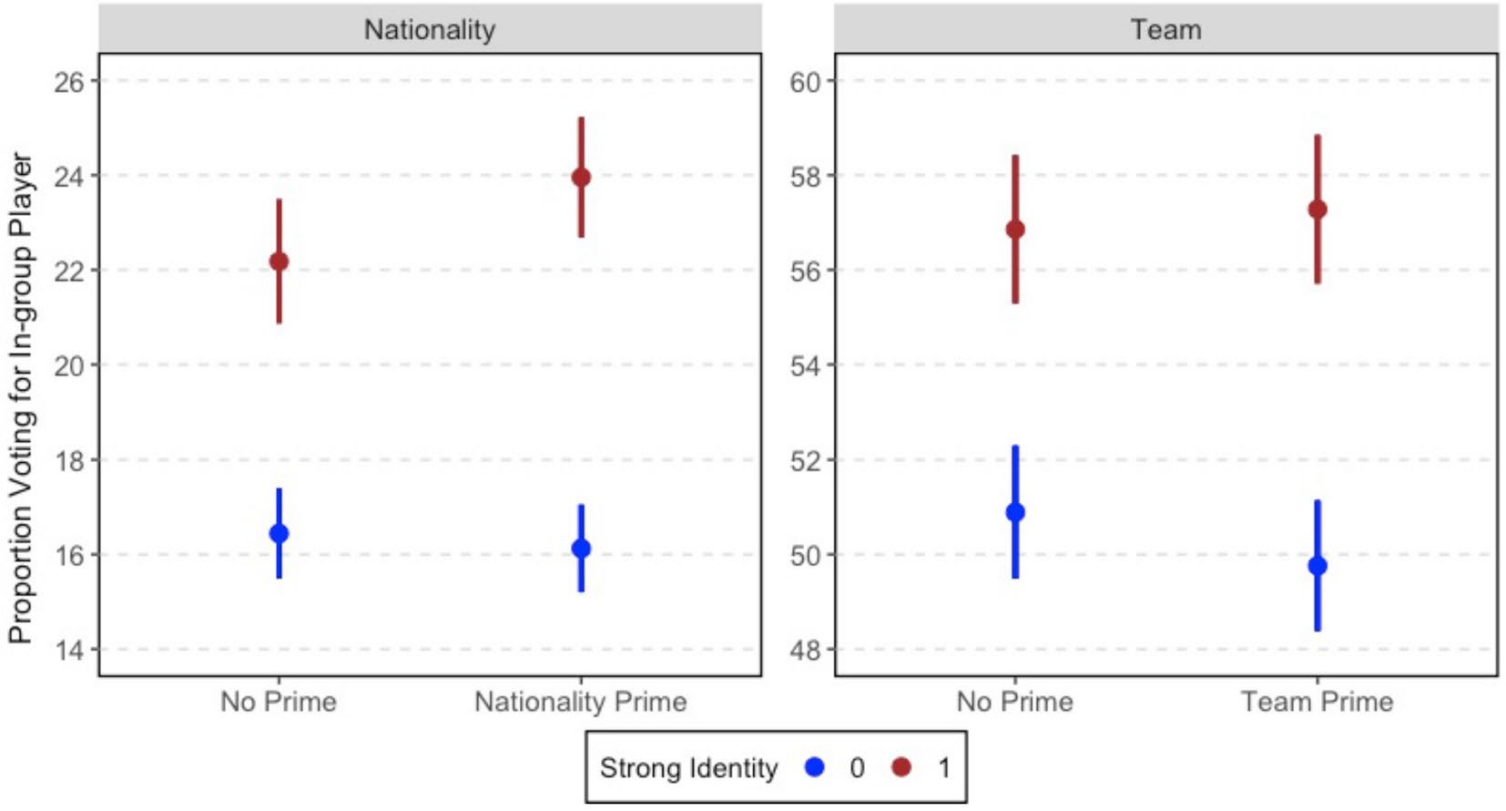
Effect of in-group primes conditional on social identity strength.

Figure 6 presents results from a test of whether the effect of the in-group prime is significantly greater among subjects who strongly identify with either their team or nationality based on the pre-treatment survey (see Fig. 5 for the survey Forza users responded to in the app).

The results in Fig. 6 come from a linear regression model in which voting for an in-group player is regressed on the treatment assignment, the binary measure of strong national/team identity, and the interaction of the two. The regression results are provided in the Supporting Information in Table S8. Also presented in the Supporting Information are results from the same regression with a continuous measure of identity strength (Table S9). Of the subjects who voted in the POS poll, approximately 23% also completed the survey ($N = 93{,}105$).

It is clear from the figure that support for in-group players is higher among strong identifiers. In the baseline condition (no prime), voting for in-group players among those who strongly identify with their nationality or team is nearly 6% points higher than among those who identify more weakly.

Based on subjects who shared the same national identity as a POS candidate, Fig. 6 shows that the effect of the nationality prime depends on the strength of their national identity. Among those with a weaker national identity (in blue), the prime had no effect on whether they voted for an in-group candidate. In contrast, the prime increased in-group voting among subjects with a strong national identity (red). Compared to those with weaker identities, priming national identity increased in-group voting among strong identifiers by 2.09% points ($p=0.07$). Turning to subjects who support the team a POS candidate played for, we do not find any evidence that the effect of the team prime differs based on strength of subjects’ attachment to their favorite team.

### Out-group derogation

**Figure 7:**
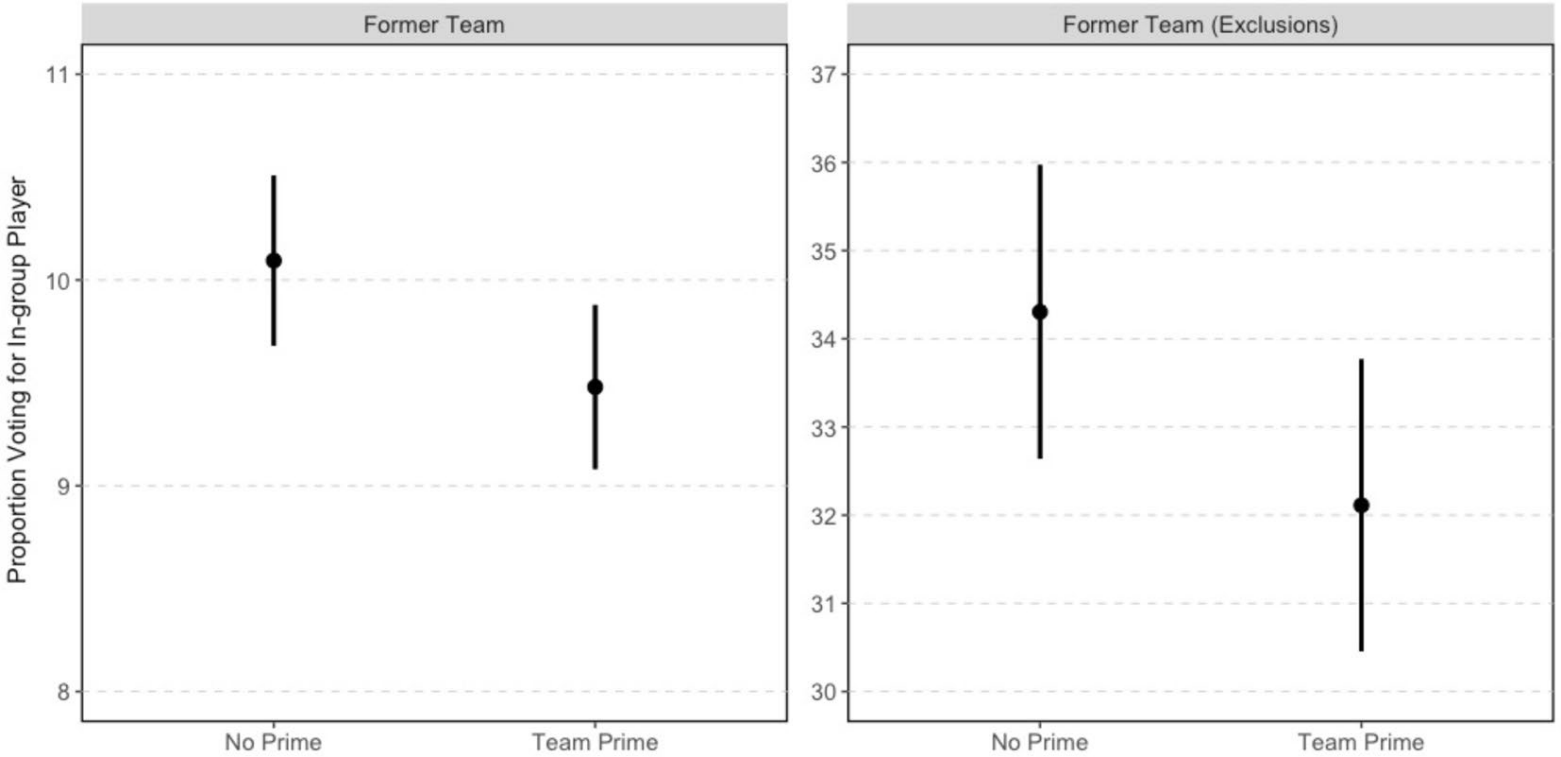
Effect of out-group prime.

To evaluate out-group derogation, we compare voting for players that have moved from the club users support to the club they played for at the time of the POS poll (listed in Table 1). (This is a slightly modified hypothesis compared to the one we preregistered about supporters from rival teams having more antipathy toward players from those out-groups. However, the analysis we present here is in line with the underlying motivation for the registered hypothesis.) For example, Kevin De Bruyne moved from VfL Wolfsburg of the German Bundesliga to his current team of Manchester City. Hence, for Wolfsburg supporters, De Bruyne used to be part of the in-group but has since become a member of the out-group. Here we restrict the analysis to users in the team condition who support a previous club of the ten POS candidates ($N = 41,137$). Figure 7 displays the mean level of voting and 95% confidence intervals from *t*-tests (all reported *p*-values are two-tailed).

Based on our test of out-group derogation, the team prime decreased voting for now out-group players by 0.61% points ($p=0.04$). That is, when presented with a ballot making salient the fact a player who used to be in an individual’s in-group is now in their out-group, individuals are less likely to vote for the player. The team prime caused a 6.1% decrease in voting for an out-group player compared to receiving no prime.

As is evident when looking at the players and clubs in the POS, there are three cases where one player’s former club is the current club of another player. This is a concern when drawing inferences about out-group derogation in this context. For example, in the case of Manchester United supporters, perhaps they voted *for* current Manchester United player David de Gea (an in-group candidate for them in the team condition), rather than *against* former Manchester United (current Real Madrid) player Cristiano Ronaldo, who would be seen as part of the out-group in the team condition where users see the club affiliation of players. Similarly Barcelona supporters might have voted for current player Lionel Messi rather than against former player Neymar and Atletico Madrid supporters may have voted for current player Antoine Griezmann rather than against former Atletico player David de Gea.

Thus, we cannot separate in-group versus out-group considerations for these three cases. However, the left panel of Fig. 7 shows the team prime did not significantly increase support for de Gea, Messi, or Griezmann. As a robustness check, in the right hand panel of Fig. 7 we excluded supporters of each of these three clubs ($N = 6,180$). The team prime decreased voting for now out-group players in the restricted sample by 2.2% points ($p=0.07$). This translates into a 6.4% decrease in voting for an out-group player compared to receiving no prime.

## Discussion

Based on a large field experiment conducted among users of a popular football live score app, we find that priming shared national identity prompted in-group favoritism in a vote to select the best player in the world. We also find evidence of out-group derogation based on analysis of voting for players who *used to be* members of an individual’s in-group but have since moved to the out-group. Further, the effect of the shared national identity is greatest among those with the strongest national identity. Priming shared and unshared team identity had no significant effect. This makes sense since for Forza users, team is already a highly salient identity whereas nationality is not. In a follow-up survey we conducted among app users, 70.2% said the team a player is a member of is very important to them compared to only 16.8% saying a player’s nationality is very important. A counter argument is that it may be the novelty of the nationality information mobilized users who otherwise would not have voted, rather than priming them with information causing their votes to change, and that this mobilization did not occur in the team condition. This, however, seems unlikely since participation in the two conditions is nearly identical (33.7% voted in the nationality condition and 33.8% in the team condition). A proportion test confirms there is no difference ($p=0.34$ for the nation and team proportions).

Our findings corroborate a burgeoning literature that has utilized sports figures and events to demonstrate in-group favoritism and discrimination against out-group members on the basis of national^30–34^, racial/ethnic^35,36^ and team identity^37^. Scholars have also demonstrated that by raising the salience of national identity, sporting events influence behaviors outside of the context of sports. Recent work has shown that success in international sporting events increased trust^38^, a willingness to cooperate^39^ and the favorability of market transactions^40^ among co-nationals.

Our field experiment improves on earlier work in at least two important respects. First, rather than studying in-group and out-group biases in a low-information environment (e.g. fictitious job applicants), the POS poll asks a group of highly knowledgeable football fans to choose among the most recognizable players in the world. Based on the follow-up survey mentioned above, 81.7% of users reported frequently watching football on TV or via an online streaming service, 88.5% reported frequently checking football scores and news in the app and on social media, TV, and in newspapers, and 76.6% follow their favorite football players on at least one social media platform. Second, our experiment was conducted on 405,179 participants from 35 countries, helping to establish its external validity. Most studies of social identity have been conducted in a single market or country among a relatively small number of participants.

The literature reporting on studies of the effects of social identity, group affinity and similar concepts on voting and other behaviors is large and impressive [see^41–43^, for reviews]. The vast majority of these studies report findings from lab experiments. While lab experiments are valuable in many ways, there is a worry across the social sciences about how well results from these studies travel outside the lab. In economics^44^ have argued for caution in extrapolating from the lab to the field because lab settings are systematically different from the “real world” on the very dimensions that are most likely to affect the choices people make (i.e. the behavior under investigation). ^45^ and^46^make a similar case about the many studies of campaign effects and political persuasion, on the one hand, and studies in social psychology on contact theory on the other hand, conducted in the lab and their often poor translation to the field. While some [see, for example^47^], take a more optimistic view, the concern about lab results not generalizing to more natural settings ought to be taken seriously. Even if we can learn much from the lab in some circumstances, the scale of the study we report in this article—both in terms of sample size and in terms of cross national samples—as well as the fact it takes place in the field, makes the findings of particular interest.

The findings presented here have potential implications beyond our study context. For example, sports betting is a real world behavior with serious consequences for individuals and society—in the US, for example, about $125B has been bet on sports since sports gambling was legalized in 2018; the illegal market is many times this size^48^. Given that subtle primes showing athletes in one’s in-group or out-group change behavior, it is likely people can be swayed in their actions by appeals to these group affiliations in the context of gambling, or advertising, using athletes. In recent years sports has also become a setting for social justice campaigns, largely as a result of the Black Lives Matter movement. Athletes in sports with audiences as varied as American football, basketball, football and Formula 1 have used their platform to raise awareness or promote action on a range of social and political issues. The evidence presented in our experiment may help us better understand—and better design studies to learn about—how individual behavior on these issues can be affected by athletes’ behavior.

This study represents a difficult test of social identity theory given our subtle prime combined with the fact that Forza users closely follow players and teams, making it likely they are cognizant of group memberships they share with the POS candidate. However, this scenario closely mirrors many of the in-person and online interactions we have as part of our everyday lives. The effects of perceiving a shared or unshared group identity is likely small in any particular interaction—and as such, difficult to detect in many small scale studies. The results of our well-powered study suggests that relatively small changes in the salience of group identities can significantly alter behavior.

## Supplementary Information

Supplementary Information.

## Data Availability

The datasets used and analyzed during the current study are available from the corresponding author on reasonable request.
