# Supplementary material for: Subtle primes of in-group and out-group affiliation change votes in a large scale field experiment
Source: Sci Rep. 2022 Dec 29;12:22526. doi: 10.1038/s41598-022-26187-x (PMC9800561; doi:10.1038/s41598-022-26187-x)
Supplement: Supplementary file 1 — Supplementary Information. [file 41598_2022_26187_MOESM1_ESM.pdf]

Subtle primes of in-group and out-group affiliation change votes in a large scale field experiment
Supplementary Information

Christopher Dawes[1] and Daniel Rubenson[2,*]

[1]New York University, Wilf Family Department of Politics, New York, 10012, United States
[2]Toronto Metropolitan University, Department of Politics, Toronto, M5B 2K3, Canada
[*]rubenson@ryerson.ca

S1 Balance

The tables below report the number of observations and average subject characteristics for each experimental condition and for each cut of the data. We have included all variables for which we have data on our subjects, including their nationality, favorite club, leagues followed on the Forza app and the strength of their club and national identity as measured in our survey (see Section S2).

In Table S1 we compare the Club condition to the Baseline condition and the Nation condition to the Baseline condition, for the entire sample. For example, in row one of Table S1, the "Diff" and "P-val" columns in the Club section are reporting the comparison in the proportion of Argentinian subjects in the Club condition with those in the Baseline.

Tables S2–S7 report similar statistics for the restricted samples of subjects in each of the analyses described above. While there are a small number of statistically significant differences, we note three things: First, we would expect some differences, just by chance; second, our large sample size means we are more likely to get small p-values; and third, those differences that are statistically significant remain very small in substantive terms. We are confident that our randomization has resulted in good balance in subject characteristics across conditions and that our results are not driven by unobserved heterogeneity.

Table S1: Summary Statistics & Balance , Full Sample (N = 405179).

	**Baseline proportion**	N	**Club proportion**	N	Difference	P-value	**Nation proportion**	N	Difference	P-value
Subject nationality										
Argentina	0.0108	1421	0.0106	1457	0.0001	0.7349	0.0117	1596	-0.0009	0.0285
Belgium	0.0305	4028	0.0311	4255	-0.0006	0.3679	0.0307	4207	-0.0002	0.7596
Brazil	0.0178	2355	0.0177	2423	0.0001	0.7869	0.0179	2444	-0.0000	0.9927
Egypt	0.0046	612	0.0046	630	0.0001	0.8174	0.0049	669	-0.0002	0.3483
Spain	0.0161	2125	0.0158	2168	0.0003	0.5876	0.0156	2135	0.0005	0.2905
France	0.0144	1905	0.0146	1995	-0.0001	0.7652	0.0144	1977	-0.0000	0.9932
Great Britain	0.0675	8904	0.0685	9349	-0.0010	0.3250	0.0675	9243	-0.0000	0.9709
Italy	0.2015	26578	0.2003	27346	0.0012	0.4464	0.2057	28153	-0.0042	0.0066
Poland	0.0055	728	0.0055	763	-0.0000	0.9037	0.0056	761	-0.0000	0.8840
Portugal	0.0349	4604	0.0341	4654	0.0008	0.2317	0.0351	4798	-0.0002	0.8268
Subject club support										
Atletico Madrid	0.003	26826	0.003	27384	-0.000	0.560	0.003	27472	0.000	0.795
FC Barcelona	0.099	30613	0.097	31172	0.002	0.077	0.095	31218	0.004	0.001
Bayern Munich	0.021	25882	0.020	26592	0.001	0.119	0.019	26836	0.002	0.010
Manchester City	0.026	25213	0.025	25851	0.000	0.782	0.025	25985	0.001	0.156
Lazio Roma	0.019	21514	0.018	21912	0.001	0.227	0.019	22235	-0.000	0.963
Liverpool FC	0.057	19730	0.058	20155	-0.001	0.534	0.058	20373	-0.001	0.546
Manchester United	0.051	21437	0.052	22006	-0.000	0.827	0.051	22166	0.001	0.414
Paris Saint-Germain	0.003	19805	0.003	20221	-0.000	0.914	0.003	20466	-0.000	0.760
Real Madrid	0.066	22309	0.065	22794	0.001	0.333	0.063	22949	0.002	0.022
Tottenham Hotspur	0.018	21778	0.019	22157	-0.001	0.144	0.018	22329	-0.000	0.828
Favorite Club	0.853	112450	0.855	116640	-0.002	0.137	0.853	116762	-0.001	0.625
Subject leagues followed										
Bundesliga	0.634	70566	0.637	73730	-0.003	0.067	0.638	73756	-0.004	0.027
LaLiga	0.873	83564	0.873	86937	-0.000	0.709	0.872	87261	0.001	0.619
Ligue 1	0.535	115085	0.540	119155	-0.005	0.007	0.539	119317	-0.004	0.040
Premier League	0.873	97752	0.877	101586	-0.004	0.002	0.875	102263	-0.002	0.085
Serie A	0.741	115113	0.744	119660	-0.003	0.060	0.747	119736	-0.006	0.000
# Leagues	3.6554	128518	3.6717	133125	-0.0162	0.0045	3.6712	133473	-0.0158	0.0057
Subject club/national identity										
Club ID	12.9912	27469	12.9588	27930	0.0324	0.1345	12.9706	28259	0.0207	0.3356
Strong Club ID	0.4204	11547	0.4136	11552	0.0068	0.1067	0.4126	11661	0.0077	0.0647
National ID	12.3597	27469	12.3141	27930	0.0456	0.0746	12.3328	28259	0.0269	0.2901
Strong Nat ID	0.3613	9924	0.3598	10048	0.0015	0.7089	0.3579	10115	0.0033	0.4113
Took Survey	0.2083	27469	0.2047	27930	0.0036	0.0204	0.2065	28259	0.0018	0.2602

Table S2: Summary Statistics & Balance, Nationality Condition as an In-Group Prime (N = 109223). Voting among Co-Nationals (corresponds to left panel of *Figure 2* in main text)

	Baseline proportion	N	Nation proportion	N	Difference	P-value
Subject nationality						
Argentina	0.0267	1420	0.0285	1595	-0.0018	0.0649
Belgium	0.0756	4027	0.0751	4206	0.0005	0.7634
Brazil	0.0442	2354	0.0436	2443	0.0006	0.6514
Egypt	0.0115	611	0.0119	668	-0.0005	0.4796
Spain	0.0399	2124	0.0381	2134	0.0018	0.1329
France	0.0358	1904	0.0353	1976	0.0005	0.6860
Great Britain	0.1672	8903	0.1651	9242	0.0021	0.3566
Italy	0.4991	26577	0.5030	28152	-0.0039	0.2024
Poland	0.0137	727	0.0136	760	0.0001	0.9153
Portugal	0.0864	4603	0.0857	4797	0.0007	0.6633
Subject club support						
Has favorite club	0.8981	47825	0.8980	50263	0.0001	0.9410
Atletico Madrid	0.0028	7238	0.0023	7663	0.0005	0.1412
FC Barcelona	0.0534	7981	0.0488	8162	0.0047	0.0009
Bayern Munich	0.0070	7023	0.0066	7339	0.0004	0.4714
Manchester City	0.0212	6779	0.0210	7000	0.0002	0.7992
Lazio Roma	0.0400	7339	0.0398	7711	0.0002	0.8664
Liverpool FC	0.0334	5557	0.0324	5825	0.0010	0.3784
Manchester United	0.0283	6019	0.0257	6363	0.0026	0.0106
Paris Saint-Germain	0.0040	5614	0.0042	5922	-0.0003	0.5138
Real Madrid	0.0379	6440	0.0389	6765	-0.0009	0.4405
Tottenham Hotspur	0.0124	5758	0.0130	6041	-0.0006	0.4244
Subject leagues followed						
Bundesliga	0.5636	28740	0.5665	30316	-0.0029	0.3402
LaLiga	0.8426	30014	0.8419	31709	0.0007	0.7618
Ligue 1	0.5397	44869	0.5416	47126	-0.0019	0.5288
Premier League	0.8348	43303	0.8365	45786	-0.0018	0.4307
Serie A	0.8132	44451	0.8180	46823	-0.0048	0.0409
Number of Leagues	3.5939	51905	3.6046	54570	-0.0107	0.2426
Subject club/national identity						
Club Identity	13.0227	9863	13.0022	10405	0.0205	0.5540
Strong Club Identity	0.4089	4033	0.4126	4293	-0.0037	0.5937
National Identity	12.3039	9863	12.3002	10405	0.0036	0.9320
Strong National Identity	0.3433	3386	0.3474	3615	-0.0041	0.5370
Took Survey	0.1852	9863	0.1859	10405	-0.0007	0.7750

Table S3: Summary Statistics & Balance, Club Condition as an In-Group Prime (N = 82761). Voting among Club Supporters (corresponds to right panel of *Figure 2* in main text)

	Baseline proportion	N	Club proportion	N	Difference	P-value
Subject nationality						
Argentina	0.0064	260	0.0060	254	0.0004	0.5038
Belgium	0.0265	1082	0.0272	1143	-0.0007	0.5436
Brazil	0.0154	629	0.0143	602	0.0011	0.1863
Egypt	0.0094	384	0.0089	374	0.0005	0.4120
Spain	0.0214	873	0.0230	967	-0.0016	0.1161
France	0.0156	638	0.0160	674	-0.0004	0.6583
Great Britain	0.0993	4051	0.1021	4285	-0.0028	0.1776
Italy	0.0669	2731	0.0653	2740	0.0017	0.3367
Poland	0.0070	286	0.0068	289	0.0002	0.7715
Portugal	0.0138	564	0.0131	551	0.0007	0.3591
Subject club support						
Atletico Madrid	0.0074	7396	0.0078	7555	-0.0004	0.4868
FC Barcelona	0.2740	11183	0.2704	11343	0.0037	0.2346
Bayern Munich	0.0575	6452	0.0555	6763	0.0020	0.2017
Manchester City	0.0706	5783	0.0707	6022	-0.0001	0.9458
Lazio Roma	0.0511	2084	0.0496	2083	0.0014	0.3504
Liverpool FC	0.1581	300	0.1612	326	-0.0031	0.2249
Manchester United	0.1417	2007	0.1435	2177	-0.0018	0.4535
Paris Saint-Germain	0.0092	375	0.0093	392	-0.0002	0.8172
Real Madrid	0.1812	2879	0.1801	2965	0.0012	0.6631
Tottenham Hotspur	0.0492	2348	0.0519	2328	-0.0027	0.0754
Subject leagues followed						
Bundesliga	0.6626	22437	0.6639	23268	-0.0013	0.6845
LaLiga	0.9274	27038	0.9291	27854	-0.0017	0.3302
Ligue 1	0.5498	37843	0.5546	38980	-0.0048	0.1672
Premier League	0.9289	28908	0.9322	29943	-0.0033	0.0637
Serie A	0.7084	37905	0.7137	39108	-0.0053	0.0925
Number of Leagues	3.7771	40807	3.7935	41954	-0.0164	0.0800
Subject club/national identity						
Club Identity	13.1018	8733	13.0403	8886	0.0615	0.1072
Strong Club Identity	0.4467	3901	0.4360	3874	0.0107	0.1516
National Identity	12.2836	8733	12.2004	8886	0.0832	0.0737
Strong National Identity	0.3683	3216	0.3501	3111	0.0182	0.0120
Took Survey	0.2140	8733	0.2118	8886	0.0022	0.4387

Table S4: Summary Statistics & Balance, Nationality Condition as an In-Group Prime (N = 20268). Voting among Co-Nationals with High versus Low National Identity Strength. Restricted to Survey Respondents (corresponds to left panel of *Figure 3* in main text)

	Control	N	Nation Treat	N	Difference	P-value
Subject nationality						
Argentina	0.0605	597	0.0607	632	-0.0002	0.9499
Belgium	0.0911	899	0.0890	926	0.0022	0.5926
Brazil	0.0488	481	0.0461	480	0.0026	0.3777
Egypt	0.0107	106	0.0093	97	0.0014	0.3095
Spain	0.0402	396	0.0429	446	-0.0027	0.3328
France	0.0277	273	0.0257	267	0.0020	0.3729
Great Britain	0.1935	1908	0.1981	2061	-0.0046	0.4066
Italy	0.4185	4128	0.4202	4372	-0.0016	0.8121
Poland	0.0134	132	0.0138	144	-0.0005	0.7793
Portugal	0.0956	943	0.0942	980	0.0014	0.7295
Subject club support						
Atletico Madrid	0.0023	1157	0.0023	1244	0.0000	0.9745
FC Barcelona	0.0550	1372	0.0464	1341	0.0086	0.0079
Bayern Munich	0.0057	1188	0.0073	1245	-0.0016	0.1788
Manchester City	0.0245	1167	0.0226	1184	0.0019	0.4034
Lazio Roma	0.0337	1181	0.0369	1251	-0.0032	0.2377
Liverpool FC	0.0345	899	0.0363	922	-0.0018	0.5091
Manchester United	0.0322	1007	0.0299	1062	0.0023	0.3705
Paris Saint-Germain	0.0039	913	0.0036	934	0.0003	0.7231
Real Madrid	0.0311	1098	0.0362	1115	-0.0051	0.0524
Tottenham Hotspur	0.0144	929	0.0170	969	-0.0027	0.1415
Subject leagues followed						
Bundesliga	0.6441	6056	0.6564	6510	-0.0123	0.0667
LaLiga	0.8905	6353	0.8971	6830	-0.0066	0.1294
Ligue 1	0.6140	8783	0.6257	9334	-0.0116	0.0878
Premier League	0.8916	8271	0.9006	8754	-0.0090	0.0357
Serie A	0.8386	8794	0.8413	9371	-0.0027	0.5953
Number of Leagues	3.8788	9703	3.9211	10276	-0.0423	0.0295
Subject club/national identity						
Club Identity	13.0227	9863	13.0022	10405	0.0205	0.5540
Strong Club Identity	0.4089	4033	0.4126	4293	-0.0037	0.5937
National Identity	12.3039	9863	12.3002	10405	0.0036	0.9320
Strong National Identity	0.3433	3386	0.3474	3615	-0.0041	0.5370

Table S5: Summary Statistics & Balance, Club Condition as an In-Group Prime (N = 17619). Voting among Club Supporters with High versus Low Club Identity Strength. Restricted to Survey Respondents (corresponds to right panel of *Figure 3* in main text)

	Control	N	Club Treat	N	Difference	P-value
Subject nationality						
Argentina	0.0115	100	0.0116	105	-0.0001	0.9291
Belgium	0.0254	222	0.0297	266	-0.0043	0.0816
Brazil	0.0163	142	0.0138	125	0.0024	0.1883
Egypt	0.0080	70	0.0084	77	-0.0004	0.7540
Spain	0.0168	147	0.0190	171	-0.0022	0.2732
France	0.0090	79	0.0123	111	-0.0032	0.0371
Great Britain	0.0965	843	0.1040	926	-0.0075	0.0985
Italy	0.0424	370	0.0471	420	-0.0047	0.1326
Poland	0.0054	47	0.0051	47	0.0003	0.7707
Portugal	0.0127	111	0.0104	94	0.0024	0.1435
Subject club support						
Atletico Madrid	0.0077	1487	0.0084	1532	-0.0008	0.5684
FC Barcelona	0.2713	2369	0.2586	2298	0.0127	0.0569
Bayern Munich	0.0551	1436	0.0551	1489	-0.0001	0.9850
Manchester City	0.0763	1355	0.0777	1370	-0.0014	0.7297
Lazio Roma	0.0389	340	0.0413	367	-0.0024	0.4232
Liverpool FC	0.1644	67	0.1676	75	-0.0031	0.5763
Manchester United	0.1552	462	0.1542	496	0.0010	0.8568
Paris Saint-Germain	0.0080	70	0.0089	79	-0.0009	0.5259
Real Madrid	0.1703	666	0.1724	690	-0.0021	0.7073
Tottenham Hotspur	0.0529	481	0.0558	490	-0.0029	0.3934
Subject leagues followed						
Bundesliga	0.7365	5395	0.7469	5591	-0.0104	0.1152
LaLiga	0.9482	6432	0.9503	6637	-0.0020	0.5421
Ligue 1	0.6178	8281	0.6292	8444	-0.0114	0.1178
Premier League	0.9623	6762	0.9659	7007	-0.0036	0.2023
Serie A	0.7743	8404	0.7885	8583	-0.0142	0.0222
Number of Leagues	4.0392	414	4.0808	394	-0.0416	0.0225
Subject club/national identity						
Club Identity	13.1018	8733	13.0403	8886	0.0615	0.1072
Strong Club Identity	0.4467	3901	0.4360	3874	0.0107	0.1516
National Identity	12.2836	8733	12.2004	8886	0.0832	0.0737
Strong National Identity	0.3683	3216	0.3501	3111	0.0182	0.0120

Table S6: Summary Statistics & Balance, Club Condition as an Out-Group Prime (N = 41137). Voting among Club Supporters for Former Players (corresponds to left panel of Figure 4 in main text)

	Control	N	Club Treat	N	Difference	P-value
Subject nationality						
Argentina	0.0096	196	0.0092	192	0.0004	0.6685
Belgium	0.0250	509	0.0243	506	0.0006	0.6823
Brazil	0.0144	294	0.0136	284	0.0008	0.5011
Egypt	0.0051	104	0.0044	92	0.0007	0.2914
Spain	0.0271	553	0.0296	615	-0.0025	0.1313
France	0.0135	275	0.0133	276	0.0002	0.8363
Great Britain	0.0787	1605	0.0782	1623	0.0005	0.8367
Italy	0.1045	2131	0.1009	2095	0.0036	0.2296
Poland	0.0066	135	0.0058	122	0.0008	0.3088
Portugal	0.0099	202	0.0098	205	0.0001	0.9383
Subject club support						
Atletico Madrid	0.0147	300	0.0157	326	-0.0010	0.4087
FC Barcelona	0.5485	11183	0.5467	11343	0.0018	0.7090
Real Sociedad	0.0022	45	0.0023	47	-0.0001	0.9009
AS Roma	0.0714	1456	0.0672	1395	0.0042	0.0950
VfL Wolfsburg	0.0027	56	0.0035	72	-0.0007	0.1874
Borussia Dortmund	0.0431	878	0.0439	910	-0.0008	0.6933
Manchester United	0.2836	5783	0.2902	6022	-0.0066	0.1399
FC Torino	0.0337	687	0.0306	634	0.0031	0.0710
Subject leagues followed						
Bundesliga	0.6425	11070	0.6454	11464	-0.0029	0.5331
LaLiga	0.9369	13099	0.9385	13392	-0.0016	0.5075
Ligue 1	0.5430	19101	0.5525	19472	-0.0095	0.0519
Premier League	0.9044	14516	0.9119	14913	-0.0075	0.0081
Serie A	0.7120	18439	0.7187	18922	-0.0067	0.1295
Number of Leagues	3.7387	1723	3.7671	1738	-0.0284	0.0340
Subject club/national identity						
Club Identity	13.0604	4407	13.0502	4264	0.0102	0.8513
Strong Club Identity	0.4409	1943	0.4301	1834	0.0108	0.3117
National Identity	12.2759	4407	12.2474	4264	0.0285	0.6676
Strong National Identity	0.3694	1628	0.3593	1532	0.0101	0.3274
Took Survey	0.2162	4407	0.2055	4264	0.0107	0.0081

Table S7: Summary Statistics & Balance, Club Condition as an Out-Group Prime (N = 6180). Voting among Club Supporters for Former Players. Excludes supporters of Manchester United, Atletico Madrid, and Barcelona (corresponds to right panel of Figure 4 in main text)

	Control	N	Club Treat	N	Difference	P-value
Argentina	0.0016	5	0.0013	4	0.0003	0.7621
Belgium	0.0106	33	0.0108	33	-0.0002	0.9326
Brazil	0.0064	20	0.0049	15	0.0015	0.4312
Egypt	0.0003	1	0.0007	2	-0.0003	0.5530
Spain	0.0090	28	0.0105	32	-0.0015	0.5492
France	0.0051	16	0.0056	17	-0.0004	0.8149
Great Britain	0.0199	62	0.0164	50	0.0035	0.3008
Italy	0.5705	1781	0.5592	1710	0.0113	0.3713
Poland	0.0029	9	0.0039	12	-0.0010	0.4826
Portugal	0.0022	7	0.0023	7	-0.0000	0.9691
Subject club support						
Real Sociedad	0.0144	45	0.0154	47	-0.0010	0.7565
AS Roma	0.4664	1456	0.4562	1395	0.0102	0.4219
VfL Wolfsburg	0.0179	56	0.0235	72	-0.0056	0.1223
Borussia Dortmund	0.2812	878	0.2976	910	-0.0164	0.1565
FC Torino	0.2201	687	0.2073	634	0.0127	0.2224
Subject leagues followed						
Bundesliga	0.6896	1787	0.7096	1786	-0.0200	0.0865
LaLiga	0.8389	2153	0.8450	2170	-0.0061	0.5103
Ligue 1	0.5724	2619	0.5840	2584	-0.0117	0.3538
Premier League	0.8479	2870	0.8734	2811	-0.0256	0.0037
Serie A	0.9193	2647	0.9192	2671	0.0001	0.9938
Number of Leagues	3.8680	247	3.9313	234	-0.0633	0.0582
Subject club/national identity						
Club Identity	12.8929	616	12.6929	521	0.2000	0.1897
Strong Club Identity	0.3847	237	0.3378	176	0.0469	0.1005
National Identity	12.0357	616	12.1344	521	-0.0986	0.5890
Strong National Identity	0.3052	188	0.3244	169	-0.0192	0.4885
Took Survey	0.1973	616	0.1704	521	0.0269	0.0063

S2 Measuring National and Team Identity

In order to more effectively carry out this study, we wanted a measure of the strength of users' national and club identities before the Player of the Season (POS) voting began. To do this, we implemented a short survey in the Forza Football app with six questions designed to capture users' closeness to their preferred club and to their nationality.

The survey items were:

1. How strongly do you identify with your football club? [1 Not at all, 2, 3, 4, 5 Extremely]
2. When talking about your football club, how often do you say "we" instead of "they"? [1 Never, 2, 3, 4, 5 Always]
3. How important is being a supporter of your football club to you? [1 Not at all, 2, 3, 4, 5 Extremely]
4. How strongly do you identify with your nationality? [1 Not at all, 2, 3, 4, 5 Extremely]
5. When talking about your nationality, how often do you say "we" instead of "they"? [1 Never, 2, 3, 4, 5 Always]
6. How important is being your nationality to you? [1 Not at all, 2, 3, 4, 5 Extremely]

Forza users saw this survey in the app and could choose to either answer one or more of the questions or exit the survey altogether. Figure S1 shows how the survey appeared to users. The order of the questions was randomized.

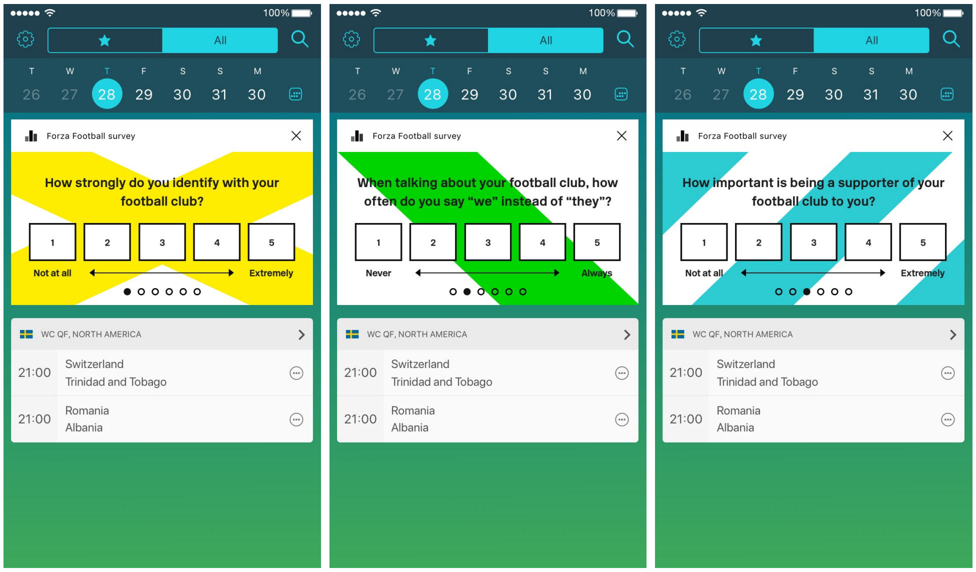

(a) Club identity (b) Club affinity (c) Club importance

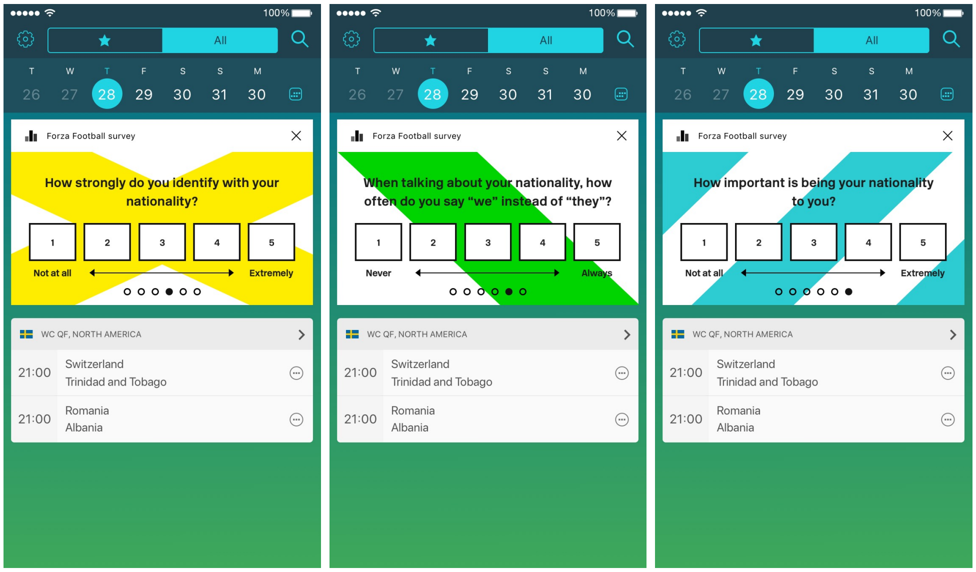

(d) National identity (e) National affinity (f) Nationality importance

Figure S1: Group identity in-app survey

S3 International and Club Comparisons

In order to test whether the results presented in *Figure 3* of the main text are not driven by a particular country or group of club supporters, we estimated the treatment effect leaving each country or each set of club supporters out of the analysis. The estimates are presented in Figures S2 and S3. The blue line in each figure represents the treatment effects reported in main text based on the entire sample. It is clear from the figures that no single country or club is driving the results.

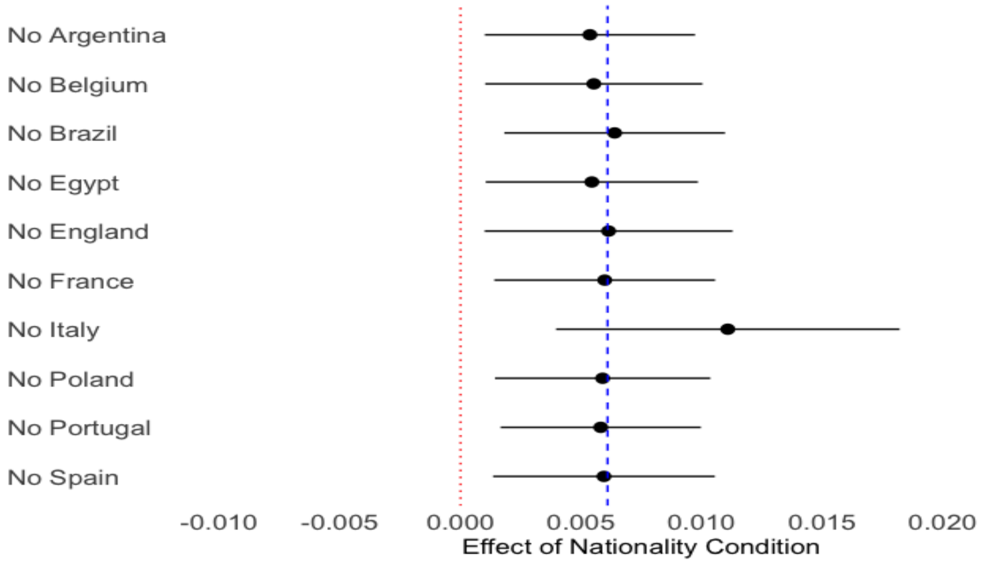

Figure S2: Estimated Treatment Effects When Excluding Users From Each Country from the Analysis. The dots represent the estimated treatment effect and the lines represent 95% Confidence Intervals. The blue line represents the estimated treatment effect based on the entire sample.

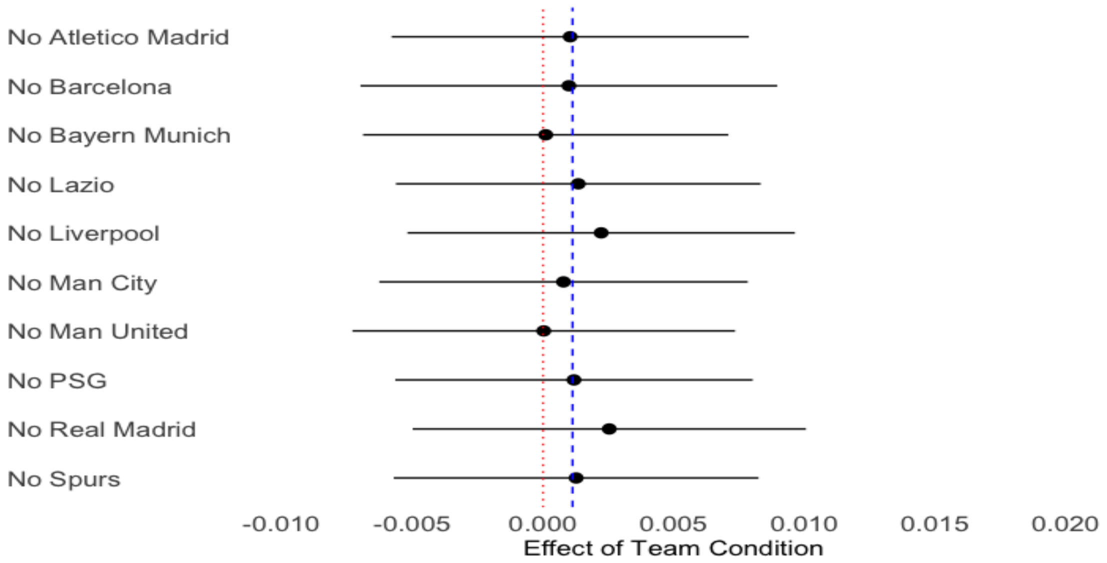

Figure S3: Estimated Treatment Effects When Excluding Users Supporting Each Club from the Analysis. The dots represent the estimated treatment effect and the lines represent 95% Confidence Intervals. The blue line represents the estimated treatment effect based on the entire sample.

S4 Regression Results

Tables S8, S9 and S11 contain regression results associated with Figures 3, 6 and 7 in the main text (Table S11 replicates Table S10 but with the continuous measure of identity strength.). In addition to the specifications without including control variables (represented in Figures 3, 6, and 7), we also ran specifications that include controls for the covariates in Table S2 above, to account for potential imbalances.

Table S8: Regression analysis associated with Figure 3 in the Main Text.

	Vote					
	**Nationality Condition**			**Team Condition**		
Prime	0.0061***	0.0049**	0.0056	0.0011	−0.0001	0.0011
	(0.0023)	(0.0020)	(0.0047)	(0.0035)	(0.0068)	(0.0032)
Argentina		0.0596***	0.0555***		0.0346	0.0562***
		(0.0076)	(0.0130)		(0.0321)	(0.0202)
Belgium		−0.2811***	−0.2705***		0.0493**	0.0083
		(0.0053)	(0.0113)		(0.0209)	(0.0099)
Brazil		−0.4997***	−0.5290***		−0.1057***	−0.0966***
		(0.0065)	(0.0144)		(0.0280)	(0.0131)
Egypt		0.1671***	0.1870***		−0.1107***	−0.0978***
		(0.0109)	(0.0261)		(0.0376)	(0.0167)
Spain		−0.5668***	−0.5655***		−0.0917***	−0.0745***
		(0.0066)	(0.0145)		(0.0258)	(0.0109)
France		−0.5481***	−0.5588***		−0.0985***	−0.0586***
		(0.0069)	(0.0172)		(0.0335)	(0.0129)
Great Britain		−0.5604***	−0.5593***		−0.0181	−0.0213***
		(0.0045)	(0.0098)		(0.0117)	(0.0054)
Italy		−0.5000***	−0.5014***		−0.0635**	−0.1175***
		(0.0040)	(0.0088)		(0.0278)	(0.0101)
Poland		−0.3380***	−0.3699***		−0.0292	−0.0125
		(0.0102)	(0.0233)		(0.0472)	(0.0191)
Portugal						0.0643**
						(0.0319)
Real Madrid		−0.0303***	−0.0238*		0.3740***	0.3943***
		(0.0055)	(0.0135)		(0.0168)	(0.0078)
FC Barcelona		−0.0614***	−0.0459***		0.5155***	0.5040***
		(0.0049)	(0.0114)		(0.0161)	(0.0075)
Liverpool FC		−0.0250***	−0.0292**		0.6476***	0.6641***
		(0.0062)	(0.0137)		(0.0171)	(0.0079)
Manchester United		−0.0240***	−0.0201		0.1706***	0.1736***
		(0.0067)	(0.0144)		(0.0172)	(0.0081)
Lazio Roma		0.1845***	0.1623***		0.1176***	0.2231***
		(0.0053)	(0.0131)		(0.0325)	(0.0132)
Athletico Madrid		−0.0110	−0.0401		0.0888**	0.0831***
		(0.0206)	(0.0494)		(0.0406)	(0.0195)
Tottenham Hotspur		0.2354***	0.2487***		0.1292***	0.1512***
		(0.0094)	(0.0197)		(0.0208)	(0.0099)
Paris Saint-Germain		−0.0084	−0.0401		−0.0341	−0.0042
		(0.0163)	(0.0394)		(0.0403)	(0.0181)
Manchester City		0.0474***	0.0428***		0.1695***	0.1560***
		(0.0073)	(0.0161)		(0.0193)	(0.0091)
Bayern Munich		−0.0032	−0.0077			
		(0.0128)	(0.0300)			
Ligue 1		−0.0056**	−0.0129**		0.0027	−0.0115***
		(0.0026)	(0.0062)		(0.0084)	(0.0039)
Bundesliga		−0.0215***	−0.0227***		−0.0155*	−0.0039
		(0.0027)	(0.0064)		(0.0094)	(0.0042)
La Liga		−0.0204***	−0.0205**		−0.0865***	−0.0976***
		(0.0034)	(0.0090)		(0.0168)	(0.0067)
Serie A		−0.0280***	−0.0286***		−0.0334***	−0.0098**
		(0.0035)	(0.0082)		(0.0099)	(0.0044)
Premier League		−0.0321***	−0.0354***		−0.0253	−0.0049
		(0.0034)	(0.0091)		(0.0193)	(0.0068)
Club Identity			−0.0024**			0.0165***
			(0.0010)			(0.0014)
National Identity			0.0067***			−0.0056***
			(0.0008)			(0.0011)
Constant	0.1659***	0.6753***	0.6340***	0.5202***	0.1751***	0.2759***
	(0.0016)	(0.0049)	(0.0189)	(0.0025)	(0.0330)	(0.0105)
N	109223	98088	18490	82761	82759	17617

***p < .01; **p < .05; *p < .1

Table S9: Regression analysis associated with Figure 6 in the main text.

	**Vote**			
	**Nationality Condition**		**Team Condition**	
Prime	−0.0032	0.0046	−0.0113	−0.0056
	(0.0067)	(0.0058)	(0.0100)	(0.0091)
High ID	0.0574***	0.0311***	0.0597***	0.0486***
	(0.0082)	(0.0072)	(0.0107)	(0.0097)
Prime * High ID	0.0209*	0.0030	0.0155	0.0124
	(0.0115)	(0.0100)	(0.0151)	(0.0137)
Argentina		0.0528***		0.0321
		(0.0130)		(0.0321)
Belgium		−0.2711***		0.0476**
		(0.0113)		(0.0210)
Brazil		−0.5311***		−0.1029***
		(0.0145)		(0.0281)
Egypt		0.1839***		−0.1134***
		(0.0261)		(0.0377)
Spain		−0.5704***		−0.0896***
		(0.0145)		(0.0259)
France		−0.5618***		−0.0993***
		(0.0172)		(0.0336)
Great Britain		−0.5640***		−0.0156
		(0.0098)		(0.0117)
Italy		−0.5026***		−0.0629**
		(0.0088)		(0.0279)
Poland		−0.3697***		−0.0383
		(0.0233)		(0.0473)
Portugal				0.0606*
				(0.0320)
Real Madrid		−0.0234*		0.3793***
		(0.0135)		(0.0169)
FC Barcelona		−0.0468***		0.5214***
		(0.0114)		(0.0161)
Liverpool FC		−0.0309**		0.6569***
		(0.0137)		(0.0171)
Manchester United		−0.0198		0.1773***
		(0.0144)		(0.0172)
Lazio Roma		0.1617***		0.1270***
		(0.0131)		(0.0326)
Athletico Madrid		−0.0413		0.0960**
		(0.0494)		(0.0407)
Tottenham Hotspur		0.2486***		0.1367***
		(0.0197)		(0.0209)
Paris Saint-Germain		−0.0401		−0.0296
		(0.0394)		(0.0404)
Manchester City		0.0437***		0.1740***
		(0.0161)		(0.0193)
Bayern Munich		−0.0074		
		(0.0300)		
League 1		−0.0127**		0.0030
		(0.0062)		(0.0084)
Bundesliga		−0.0233***		−0.0136
		(0.0064)		(0.0094)
La Liga		−0.0211**		−0.0854***
		(0.0090)		(0.0168)
Serie A		−0.0289***		−0.0326***
		(0.0082)		(0.0100)
Premier League		−0.0357***		−0.0235
		(0.0091)		(0.0193)
Constant	0.1644***	0.6769***	0.5089***	0.2904***
	(0.0048)	(0.0124)	(0.0072)	(0.0269)
N	20268	18490	17619	17617

***p < .01; **p < .05; *p < .1

Table S10: Regression analysis replicating the analysis in Table S9 using a continuous measure of identity.

	Vote			
	**Nationality Condition**		**Team Condition**	
Prime	−0.0190	−0.0003	−0.0209	−0.0094
	(0.0228)	(0.0199)	(0.0394)	(0.0357)
ID	0.0129***	0.0059***	0.0169***	0.0144***
	(0.0013)	(0.0011)	(0.0021)	(0.0019)
Prime * ID	0.0019	0.0005	0.0013	0.0007
	(0.0018)	(0.0016)	(0.0030)	(0.0027)
Argentina		0.0537***		0.0311
		(0.0130)		(0.0321)
Belgium		−0.2694***		0.0493**
		(0.0113)		(0.0209)
Brazil		−0.5298***		−0.1053***
		(0.0144)		(0.0280)
Egypt		0.1868***		−0.1149***
		(0.0261)		(0.0377)
Spain		−0.5659***		−0.0914***
		(0.0145)		(0.0259)
France		−0.5591***		−0.0975***
		(0.0172)		(0.0336)
Great Britain		−0.5601***		−0.0168
		(0.0098)		(0.0117)
Italy		−0.5008***		−0.0611**
		(0.0088)		(0.0279)
Poland		−0.3675***		−0.0307
		(0.0233)		(0.0472)
Portugal				0.0617*
				(0.0319)
Real Madrid		−0.0234*		0.3766***
		(0.0135)		(0.0168)
FC Barcelona		−0.0455***		0.5187***
		(0.0114)		(0.0161)
Liverpool FC		−0.0297**		0.6525***
		(0.0137)		(0.0171)
Manchester United		−0.0200		0.1736***
		(0.0144)		(0.0172)
Lazio Roma		0.1614***		0.1200***
		(0.0131)		(0.0326)
Athletico Madrid		−0.0405		0.0962**
		(0.0494)		(0.0406)
Tottenham Hotspur		0.2483***		0.1339***
		(0.0197)		(0.0208)
Paris Saint-Germain		−0.0387		−0.0323
		(0.0394)		(0.0403)
Manchester City		0.0434***		0.1732***
		(0.0161)		(0.0193)
Bayern Munich		−0.0075		
		(0.0300)		
League 1		−0.0128**		0.0031
		(0.0062)		(0.0084)
Bundesliga		−0.0231***		−0.0144
		(0.0064)		(0.0094)
La Liga		−0.0210**		−0.0870***
		(0.0090)		(0.0168)
Serie A		−0.0291***		−0.0331***
		(0.0082)		(0.0099)
Premier League		−0.0358***		−0.0235
		(0.0091)		(0.0193)
Constant	0.0251	0.6124***	0.3145***	0.1284***
	(0.0164)	(0.0188)	(0.0283)	(0.0360)
N	20268	18490	17619	17617

***$p < .01$; **$p < .05$; *$p < .1$

Figure S4a plots the effect of the nationality in-group prime conditional on national identity strength and Figure S4b plots the effect of the team in-group prime conditional on team identity strength based on Table S10. (We use the R package interplot [1].) Here we are we using the continuous measure of strength of identity as opposed to the dichotomized measure reported in the main text. The interaction between the in-group prime and the continuous measure of identity is not significant in either regression, but Figure S4 shows that the effect of the nationality prime is nearly significant (at conventional levels) among users with strong national identity.

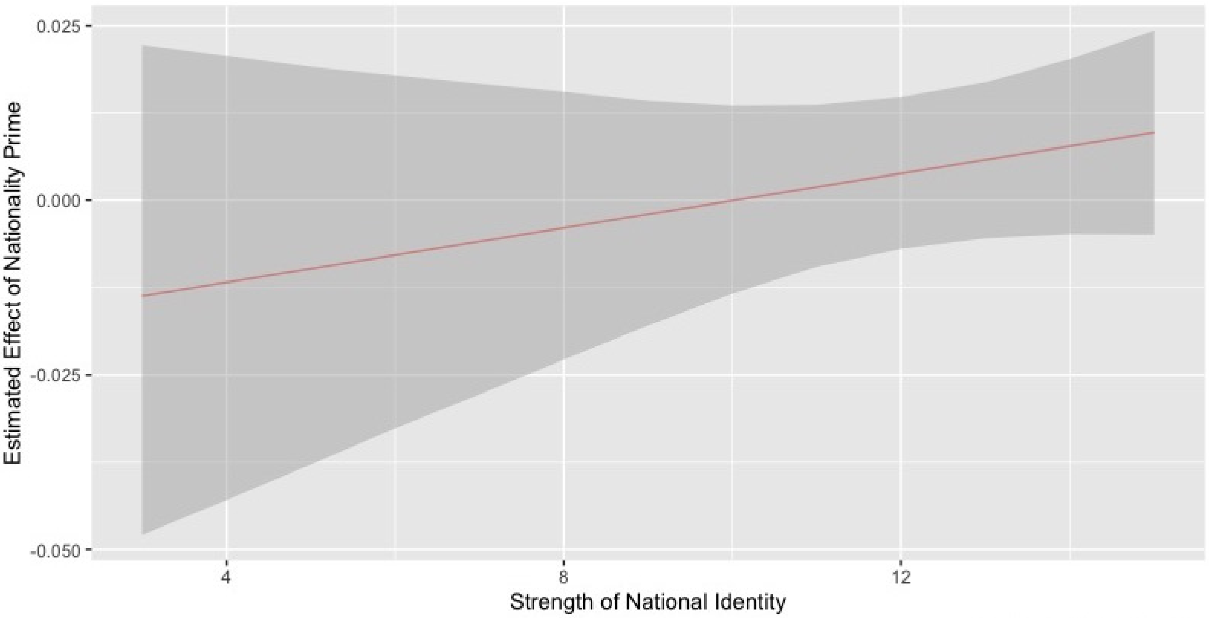

(a) Effect of In-group Prime Conditional on National Identity Strength

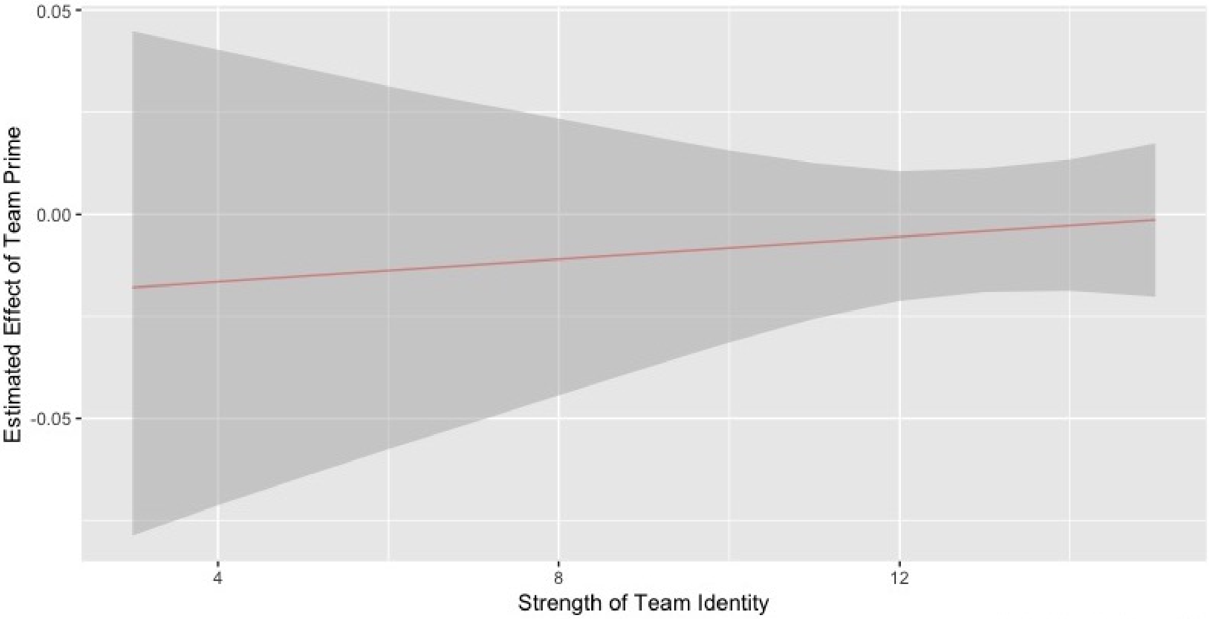

(b) Effect of In-group Prime Conditional on Team Identity Strength

Figure S4: Heterogeneous Effects of Identity Strength, In-group Prime

Table S11: Regression analysis associated with Figure 7 in the main text.

	Vote					
	Team Condition			Team Condition Excluding FC Barcelona, Manchester United & Atletico Madrid		
Prime	−0.0061**	−0.0040	−0.0143***	−0.0219*	−0.0153	−0.0479**
	(0.0029)	(0.0025)	(0.0052)	(0.0120)	(0.0094)	(0.0217)
Argentina		−0.0212	−0.0150		−0.0546	−0.0516
		(0.0129)	(0.0196)		(0.1237)	(0.1631)
Belgium		−0.0067	−0.0226		−0.0073	0.0439
		(0.0081)	(0.0167)		(0.0465)	(0.0957)
Brazil		0.0710***	0.0707***		−0.0181	−0.0373
		(0.0106)	(0.0214)		(0.0631)	(0.1220)
Egypt		−0.0296	−0.0295		0.0760	0.3844
		(0.0181)	(0.0385)		(0.2139)	(0.3623)
Spain		0.0021	0.0149		0.0420	0.1989
		(0.0077)	(0.0176)		(0.0530)	(0.1391)
France		0.0110	0.0225		−0.0179	−0.0369
		(0.0109)	(0.0282)		(0.0652)	(0.1298)
Great Britain		−0.0080*	0.0042		0.0079	−0.0276
		(0.0048)	(0.0102)		(0.0361)	(0.0777)
Italy		0.0130*	0.0060		0.0268*	0.0057
		(0.0069)	(0.0173)		(0.0152)	(0.0328)
Poland		0.0096	0.0375		0.1616**	0.0396
		(0.0158)	(0.0367)		(0.0811)	(0.3622)
Manchester United		0.1161***	0.1197***			
		(0.0105)	(0.0211)			
AS Roma		0.6189***	0.6423***		0.5979***	0.6436***
		(0.0122)	(0.0257)		(0.0168)	(0.0352)
Torino FC		0.0798***	0.0934***		0.0578***	0.0936**
		(0.0136)	(0.0306)		(0.0197)	(0.0443)
Real Sociedad		0.0420	0.0581		0.0368	0.0201
		(0.0282)	(0.0660)		(0.0444)	(0.1026)
FC Barcelona		−0.0011	0.0096			
		(0.0103)	(0.0207)			
VfL Wolfsburg		0.0341	0.0062		0.0142	−0.0097
		(0.0245)	(0.0557)		(0.0340)	(0.0797)
Borussia Dortmund		0.0165	0.0166			
		(0.0118)	(0.0243)			
League 1		0.0052*	0.0059		−0.0092	−0.0326
		(0.0031)	(0.0064)		(0.0119)	(0.0282)
Bundesliga		−0.0061*	−0.0089		0.0143	0.0497
		(0.0032)	(0.0071)		(0.0138)	(0.0336)
La Liga		0.0245***	0.0289**		−0.0463***	−0.0505
		(0.0056)	(0.0139)		(0.0156)	(0.0402)
Serie A		0.0030	0.0109		0.0068	0.0339
		(0.0034)	(0.0076)		(0.0208)	(0.0508)
Premier League		−0.0112**	−0.0112		0.0084	−0.0014
		(0.0048)	(0.0133)		(0.0165)	(0.0454)
Club Identity			0.0013			−0.0068
			(0.0011)			(0.0045)
National Identity			0.0002			0.0012
			(0.0009)			(0.0036)
Constant	0.1009***	0.0056	−0.0324	0.3430***	0.0556**	0.1011
N	41137	41136	8670	6180	6180	1137

***p < .01; **p < .05; *p < .1

S5 Follow Up Survey Questions

We fielded a survey among a sample of 21116 *Forza Football* users in December 2019. The question wordings for the items we use in the paper are:

1. *Thinking of your favorite football players, how important are the following?* [Not at all important; A little important; Moderately important; Very important; Extremely important]
   - Their performance on the pitch
   - The club they play for
   - Their nationality
   - Their involvement in social causes
   - Their activity on social media
   - Their engagement with fans
2. *How often do you do the following?* [Never;Rarely; Occasionally; Frequently]
   - Watch football matches on TV or via a streaming service
   - Attend football matches in person
   - Follow football scores and news on social media, TV, newspapers, etc.
   - Buy club merchandise (e.g. clothing)
3. *On which social media platforms do you follow your favorite football players? (Select all that apply)*
   - Twitter
   - Facebook
   - Instagram
   - Tumblr
   - Pintrest
   - Snapchat
   - Other
   - None

References

[1] Solt, F. & Hu, Y. interplot: Plot the effects of variables in interaction terms. available at the comprehensive r archive network (cran) (2015).
